# Supplementary material for: New Water-Soluble Poly(propylene imine) Dendrimer Modified with 4-Sulfo-1,8-naphthalimide Units: Sensing Properties and Logic Gates Mimicking
Source: Sensors (Basel). 2023 Jun 1;23(11):5268. doi: 10.3390/s23115268 (PMC10256094; doi:10.3390/s23115268)
Supplement: Supplementary file 1 [file sensors-23-05268-s001.zip › sensors-2398540-supplementary.pdf]

sensors

MDPI

Supporting Information

New Water Soluble Poly(propylene imine) Dendrimer modified with 4-sulfo-1,8-naphthalimide units: Sensing properties and Logic Gates Mimicking

Awad I. Said[1,2*], Desislava Staneva[3], Ivo Grabchev[1*]

[1] Sofia University “St. Kliment Ohridski”, Faculty of Medicine, 1407 Sofia, Bulgaria; i.grabchev@chem.uni-sofia.bg (I.G.)

[2] Assiut University, Faculty of Science, Department of Chemistry 71516 Assiut, Egypt; awadsaid@aun.edu.eg

[3] University of Chemical Technology and Metallurgy, Department of Textile, Leader, and Fuels, 1756 Sofia, Bulgaria; grabcheva@mail.bg

* Correspondence: ; awadsaid@aun.edu.eg (A.S.) i.grabchev@chem.uni-sofia.bg (I.G.),

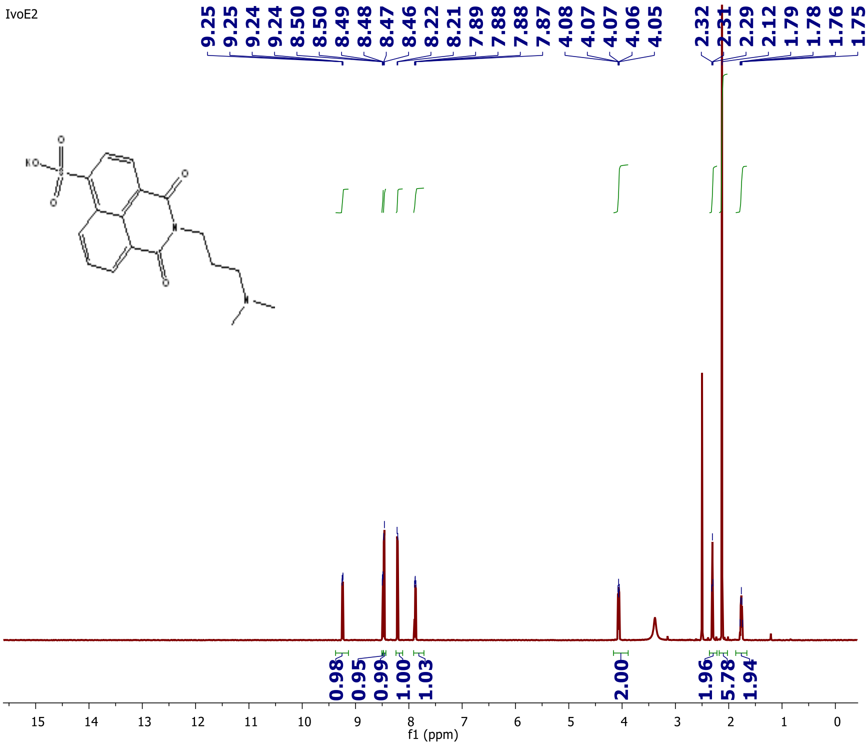

	Parameter	Value
1	Data File Name	E:/ after/ prof. Ivo/ Prof. Ivo & Awad 2021/ NMR/ Ivo_NMR_21 B/ IvoE2/ 43/ pdata/ 1/ 1r
2	Title	IvoE2
3	Comment	
4	Origin	Bruker BioSpin GmbH
5	Owner	miro
6	Site	
7	Spectrometer	spect
8	Author	
9	Solvent	DMSO
10	Temperature	293.0
11	Pulse Sequence	zg30
12	Number of Scans	1
13	Receiver Gain	128
14	Relaxation Delay	1.0000
15	Pulse Width	10.8000
16	Acquisition Time	1.7039
17	Acquisition Date	2021-12-03T00:14:10
18	Modification Date	2021-12-03T15:36:18
19	Spectrometer Frequency	599.98
20	Spectral Width	9615.4
21	Lowest Frequency	-252.0
22	Nucleus	1H
23	Acquired Size	16384
24	Spectral Size	65536

Figure S1. ^{1}H-NMR of the monomer SNIM.

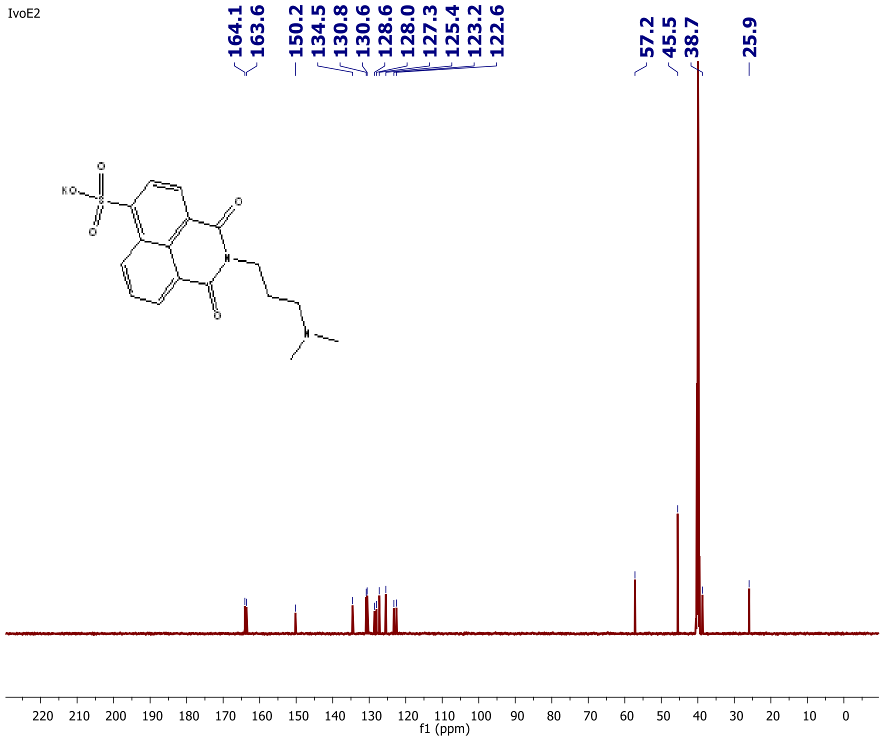

	Parameter	Value
1	Data File Name	E:/ after/ prof. Ivo/ Prof. Ivo & Awad 2021/ NMR/ Ivo_NMR_21 B/ IvoE2/ 45/ pdata/ 1/ 1r
2	Title	IvoE2
3	Comment	
4	Origin	Bruker BioSpin GmbH
5	Owner	miro
6	Site	
7	Spectrometer	spect
8	Author	
9	Solvent	DMSO
10	Temperature	293.0
11	Pulse Sequence	zgdc30
12	Number of Scans	1024
13	Receiver Gain	2050
14	Relaxation Delay	1.5000
15	Pulse Width	9.8000
16	Acquisition Time	0.4544
17	Acquisition Date	2021-12-03T 00:51:08
18	Modification Date	2021-12-03T 15:36:20
19	Spectrometer Frequency	150.87
20	Spectral Width	36057.7
21	Lowest Frequency	-1430.9
22	Nucleus	13C
23	Acquired Size	16384
24	Spectral Size	65536

Figure S2. ^{13}C-NMR of the monomer SNIM.

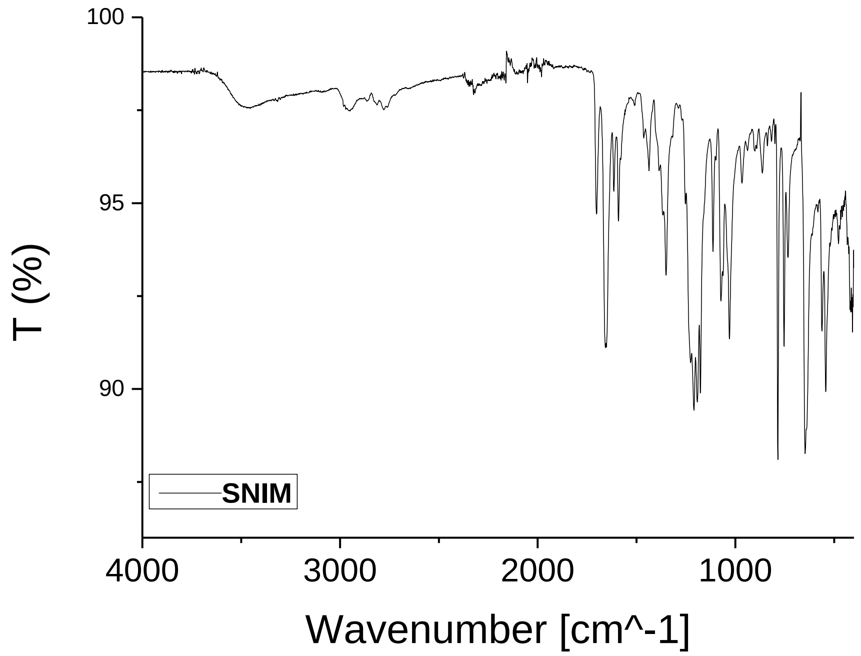

Figure S3. FTIR spectrum of the monomer SNIM.

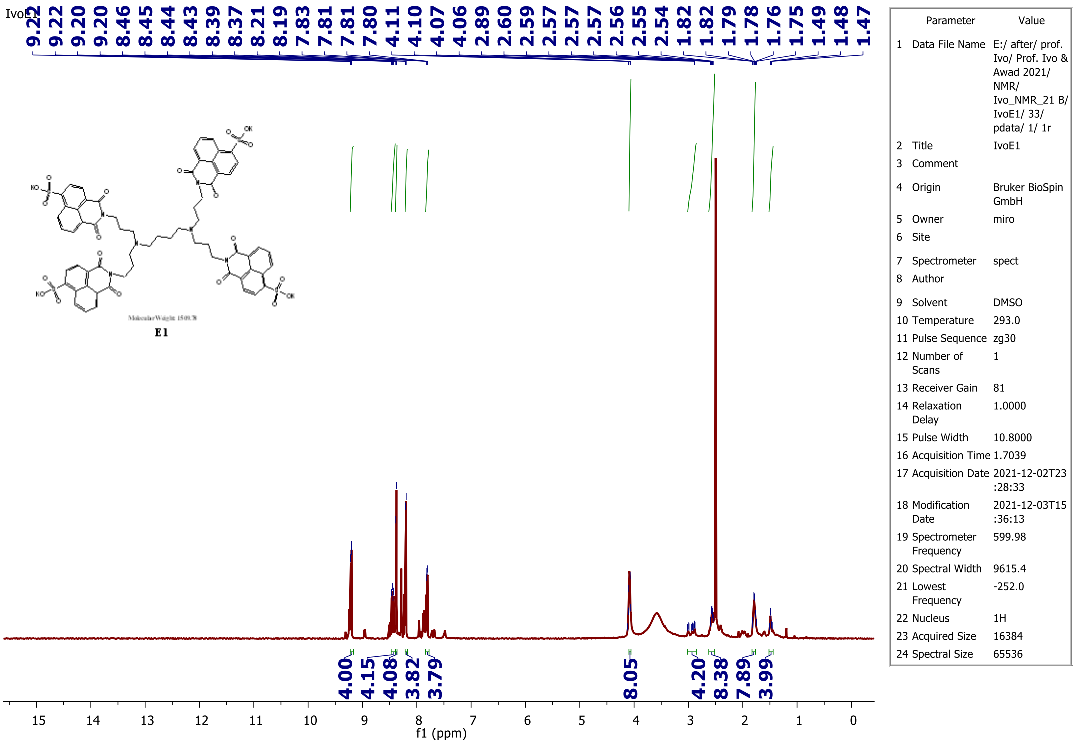

Figure S4. ^{1}H-NMR spectrum of the dendrimer SNID.

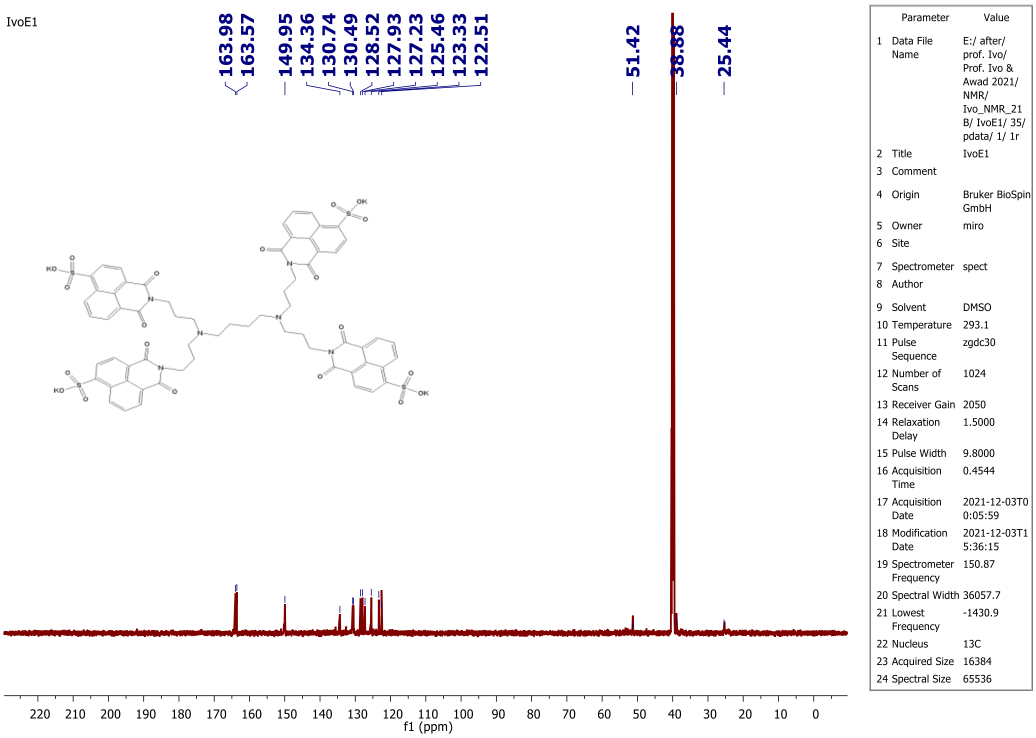

Figure S5. ^{13}C-NMR spectrum of the dendrimer SNID.

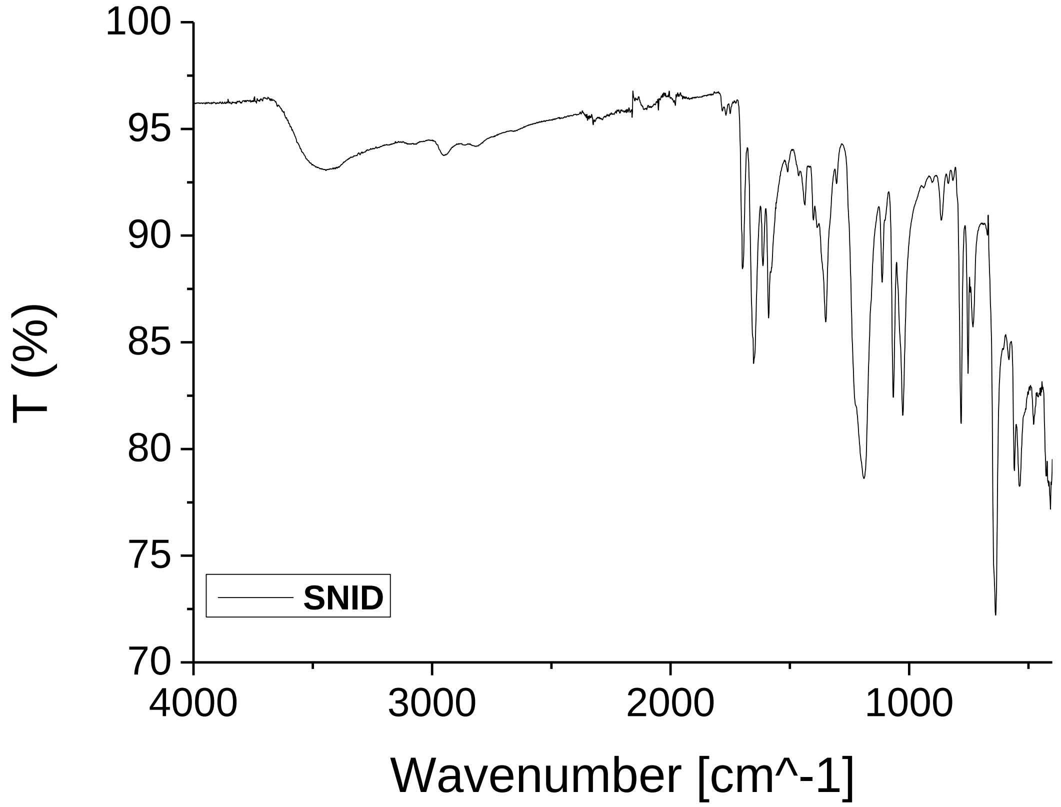

Figure S6. FTIR spectrum of the dendrimer SNID.

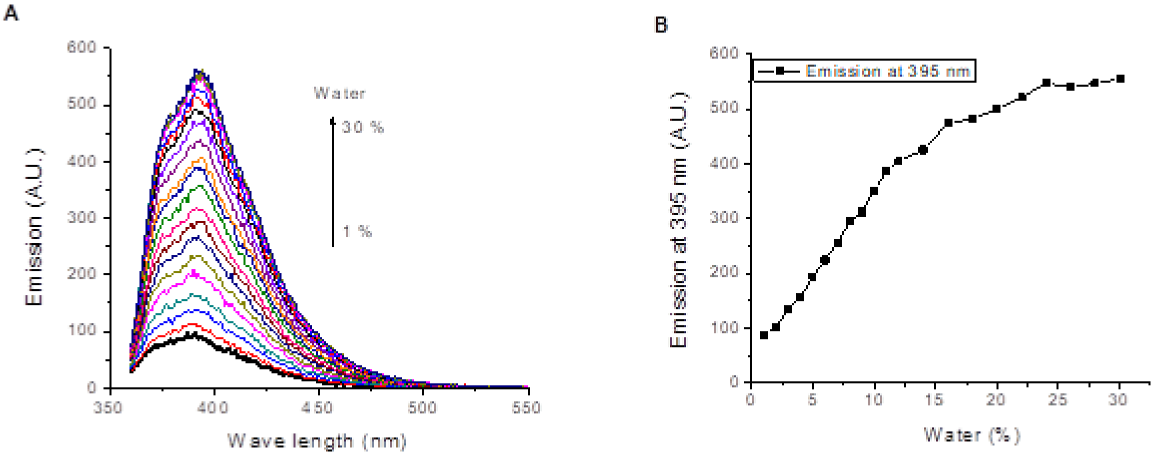

Figure S7. Influence of water on (**A**) emission spectrum and (**B**) emission at 395 nm of SNIM solution in ethanol, c = 10^{-5} M, excitation at 340 nm.
